# Characterization of endogenous pararetroviruses in yam (*Dioscorea* spp.) genomes revealed four pararetrovirus groups, including a dioscovirus-like lineage: implications for diagnostics and yam germplasm exchange

**DOI:** 10.1007/s00705-026-06703-4

**Published:** 2026-08-07

**Authors:** B. O. Osundahunsi, L. Moshood, O. A. Kolade, B. O. Odu, P. Lava Kumar

**Affiliations:** 1 International Institute of Tropical Agriculture (IITA), Oyo Road, Ibadan, Nigeria; 2 Department of Crop Production and Protection, Faculty of Agriculture, Obafemi Awolowo University, Ile-Ife, Nigeria

## Abstract

Yams (*Dioscorea* spp.) are an important vegetatively propagated food security crop grown for their starchy tubers. Yams are susceptible to several viruses, and their genomes harbor a diverse array of endogenous pararetroviral sequences (EPRVs), which complicate diagnostics and germplasm exchange because of their similarity to episomal viruses. To better characterize EPRV diversity, we analyzed 86 publicly available whole-genome sequences from five *Dioscorea* species (*D. rotundata*,* D. alata*,* D. praehensilis*,* D. abyssinica*, and *D. dumetorum*). Assembled genomes were screened for endogenous pararetrovirus sequences (EPRVs) using the CAULIFINDER pipeline, targeting conserved RT/RNase H domains of the *Caulimoviridae* family. Our analyses revealed four major EPRV groups in *D. rotundata*: Yendovirus, Badnavirus, Yam Endovirus 1, and a Dioscovirus-like lineage. While most insertions were fragmented, we found full-length putative viral genomes corresponding to the Yam Badnavirus and Dioscovirus-like clades. These findings expand knowledge of yam EPRV diversity, contribute to the development of improved diagnostic tools to differentiate endogenous and episomal forms, and promote a science-based, risk-proportionate approach to yam phytosanitation that facilitates global germplasm exchange while maintaining biosecurity.

**Supplementary Information:**

The online version contains supplementary material available at https://doi.org/10.1007/s00705-026-06703-4.

## Introduction

Yams (*Dioscorea* spp.) are vegetatively propagated crops widely grown for their starchy tubers in West Africa. Of the eight main food species, *D. rotundata* (white yam) and *D. alata* (water yam) are vital for food security and income in the West African yam belt, which stretches from Cameroon to Côte d’Ivoire [1]. However, white yam production is severely hindered by mosaic disease caused by *Yam mosaic virus* (YMV, genus *Potyvirus*). Other viruses, often latent or cryptic in West African yams, include *Yam mild mosaic virus* (YMMV, genus *Potyvirus*), Yam virus Y (YVY), and several *Badnavirus* species (family *Caulimoviridae*, genus *Badnavirus*) [2].

The family *Caulimoviridae* comprises 10 genera: *Badnavirus*,* Caulimovirus*,* Cavemovirus*,* Dioscovirus*,* Petuvirus*,* Rosadnavirus*,* Solendovirus*,* Soymovirus*,* Tungrovirus*, and *Vaccinivirus* [3, 4]. These viruses typically encode 1 to 8 open reading frames (ORFs) and produce a polyprotein containing domains such as a movement protein (MP), a virion-associated protein (VAP), an aspartic protease (AP), and a reverse transcriptase (RT) with RNase H activity (4).

Several *Badnaviruses* have been identified to infect a range of yam species, which are collectively known as Dioscorea bacilliform viruses (DBVs) [5]. To date, only a few badnavirus genomes have been characterized, including *Dioscorea bacilliform AL virus* (DBALV) and *Dioscorea bacilliform SN virus* (DBSNV). Further studies have identified high diversity among badnavirus sequences in yam germplasm, with at least ten additional tentative DBV species described based on partial reverse transcriptase (RT)-ribonuclease H sequence data [5].

*Badnaviruses* are a significant concern because their genomes can integrate into host chromosomes as endogenous viral elements, referred to as endogenous pararetroviruses (EPRVs) [3]. The discovery of endogenous badnaviral sequences in yam genomes followed the development of degenerate primers targeting badnavirus RNase H [6], which uncovered diverse badnavirus-like sequences in yams from the Southern Pacific [7]. Subsequent studies revealed the widespread presence of these sequences in *D. alata*,* D. rotundata*, and *D. cayenensis* [8–10]. The high sequence similarity between endogenous and episomal DBVs makes it difficult to distinguish them using conventional PCR-based diagnostics. Bousalem et al. [9]. demonstrated that endogenous sequences can be indistinguishable from infectious DBVs, posing a serious challenge for virus indexing and health certification [10–12].

Another challenge is the phenomenon of episomal infection from EPRVs. Although reactivation of EPRVs has not been reported in yams, studies in other crops highlight the associated risks, especially when crops are under stress or during hybridization. In banana, activation of *Banana streak virus* (BSV) sequences integrated into the B-genome of *Musa* spp. leads to episomal infections [13]. Similar reactivation has been documented in *Petunia* hybrids (*Petunia vein clearing virus*, PVCV), *Nicotiana edwardsonii* (*Tobacco vein clearing virus*, TVCV), and sugarcane (*Sugarcane bacilliform virus*, SCBV), which highlights the potential risk posed by EPRVs [13–15].

Even without reactivation, recombination between endogenous and episomal badnaviruses can generate novel genotypes. For instance, *Dioscorea bacilliform RT virus 3* (DBRTV3) is a recombinant of multiple yam badnavirus species, including *Dioscorea bacilliform SN virus*, (which occurs mainly in an endogenous form, and *Dioscorea bacilliform AL virus* (DBALV) [5]. These recombination events complicate virus identification and threaten breeding programs aimed at developing improved yam varieties. Many EPRVs occur as fragmented, rearranged, or multiple copies scattered throughout the genome, which can mask or mimic active infections [16]. Some retain near-complete open reading frames (ORFs) and regulatory elements, further complicating reliable detection of episomal viruses using PCR alone.

Next-generation sequencing (NGS) has transformed the study of plant EPRVs by enabling genome-wide characterization of integrated viral elements [16, 17]. Comparative genomic analyses have revealed extensive diversity and lineage-specific accumulation of endogenous caulimovirids in several plants [16–18]. Geering et al. [18]. used plant genomic sequences from public databases to demonstrate the widespread but uneven distribution of Florendoviruses across flowering plant genomes. Similarly, Diop et al. [17]. reported that different seed plant accessions harbor distinct sets of endogenous *Caulimovirid* RT loci, with no single viral operation taxonomic unit (OTU) shared across all host groups.

The availability of whole-genome sequences (WGS) for yams, including the first *D. rotundata* reference genome released in 2017 [19] and additional sequences from diverse cultivars and accessions, enables comprehensive screening of endogenous *Badnavirus* diversity. Given the origin and domestication history of *D. rotundata* in West Africa, genomic datasets from this region offer valuable insights into cultivar-specific EPRV diversity.

In this study, we used integrated genomic and phylogenetic approaches to characterize endogenous badnavirus diversity in West African yam production systems to enhance the understanding of their diversity and integration dynamics and support the development of robust diagnostic tools for virus indexing, thereby facilitating the safe distribution of yam germplasm.

## Materials and methods

### Genome dataset

Whole genome sequencing (WGS) data for 86 yam accessions were retrieved from the NCBI Sequence Read Archive (SRA). The dataset comprised 65 *D. rotundata* accessions, with 53 improved varieties (intraspecific hybrids) and 12 landraces, eight *D. praehensilis* accessions, five each of *D. abyssinica* and *D. dumetorum*, and three *D. alata* accessions (hybrids) (Supplementary Table S1). *Dioscorea abyssinica* and *D. praehensilis* were included as progenitor species of *D*. *rotundata*, while *D. alata* and *D. dumetorum* served as comparative groups to assess EPRV diversity across yam species. According to SRA metadata, all *D. rotundata*, *D. prahensilis*, and *D. abyssinica* samples were sequenced as paired-end short reads using random shotgun libraries. *D. alata* samples were sequenced with shotgun, whereas *D. dumetorum* samples were generated using single-end (SE) long-read sequencing.

### EPRV detection using CAULIFINDER

Raw reads were quality filtered to remove adapters and low-quality bases using BBTools (https://sourceforge.net/projects/bbmap/). Short-read datasets were assembled de novo with SPAdes v3.15.3 [20], and long-read datasets were assembled with Flye v2.9.4 [21], both using default parameters. Assembly quality was assessed with Quast v5.3 (Supplementary Table 1). EPRVs from the family *Caulimoviridae* were identified using the CAULIFINDER pipeline [22], which employs two complementary branches. Branch A reconstructs conserved viral genomes by clustering *Caulimoviridae*-derived fragments, enabling recovery of representative repeat-derived endogenous pararetroviral elements and their tentative classification into *Caulimoviridae* genera. Branch B detects EPRVs using profile-based searches of conserved RT/RNase H domains, extracting sequences with the highest similarity to *Caulimoviridae*. This strategy detects both low-copy-number and single-copy integrations not captured by repeat-based methods. To improve sensitivity in Branch A, the repeat detection threshold was adjusted by using the --hsp false flag, reducing the minimum copy number from 5 to 3 (https://hub.docker.com/r/urgi/docker_caulifinder).

Using this approach, we distinguished two categories of endogenous caulimovirid sequences: (i) EPRVs, which are sequences with *Caulimoviridae*-derived sequence retaining an intact or near-intact RT/RNase H region detectable by homology-based searches, and (ii) EPRV repeats (EPRVr), which are fragmented, repeat-derived sequences dispersed across the yam genome and identified through repeat clustering. Lineage descriptors (e.g., Badnavirus-like, Dioscovirus-like) mentioned in this study indicate phylogenetic affinity for genus-level recognition and do not imply biological activity.

### HMM profile construction and screening

Multiple sequence alignments (MSAs) of the RT/RNase H region were constructed from the representative sequences of the genera *Badnavirus*,* Yendovirus*, and *Dioscovirus* downloaded from the NCBI database. These alignments were manually curated to remove redundancy using an 80% amino acid sequence identity threshold. This cutoff was selected as an operational criterion to approximate species-level clustering within the *Caulimoviridae*, consistent with commonly used RT/RNase H–based demarcation approaches [4]. The curated MSAs were used to generate Hidden Markov model (HMM) profiles with HMMER v3.4 [23]. Also, the open reading frames (ORFs) were extracted from the CAULIFINDER Branch A sequences in both forward and reverse orientations, retaining only those encoding proteins of ≥ 500 amino acids (aa). These sequences (Branch A) were subsequently screened using the curated RT/RNAseH pHMM models. The resulting sequence hits were deduplicated using CD-HIT-EST v4.8.1 [24], and the longest representative sequences per cluster (80% sequence identity) were retained for subsequent analysis (Supplementary Table S2).

Similarly, sequences from CAULIFINDER Branch B (Supplementary file), representing putative EPRV of RT/RNase H, were pooled across all assemblies and deduplicated using CD-HIT-Protein at 80% aa sequence identity (Supplementary Table 3). The curated datasets from both branches were used for downstream evolutionary analyses.

### Phylogenetic analysis of EPRVs

To infer evolutionary relationships among yam EPRVs, maximum-likelihood (ML) phylogenies were constructed from alignments of the conserved RT/RNase H domain. Representative RT-RNase H sequences from all recognized *Caulimoviridae* genera, including *Badnavirus*,* Caulimovirus*,* Cavemovirus*,* Dioscovirus*,* Petuvirus*,* Rosadnavirus*,* Solendovirus*,* Soymovirus*,* Tungrovirus*,* Vaccinivirus*, *Ruflodivirus*, and plant EPRV genera Florendovirus, *Xendovirus*,* Yendovirus*,* Zendovirus*,* Gymnendovirus*,* Fermendovirus*, and *Wendovirus* were included [17, 25] (Supplementary Table S4).

Multiple sequence alignments (MSAs) were generated with MAFFT v7 [26] and manually trimmed to remove ambiguously aligned regions. To assess the robustness of inferred EPRVs diversity, phylogenetic analysis was conducted using a non-redundant dataset clustered at 80% sequence similarity. ML trees were reconstructed separately for CAULIFINDER Branch A and B outputs to evaluate evolutionary proximity to known EPRVs and episomal *Caulimoviridae* viruses. Final phylogenies were inferred in IQ-TREE v2.0 [27], with the optimal nucleotide substitution model selected by ModelFinder [28] and branch support estimated using 1,000 ultrafast bootstrap replicates (UFBoot). To reduce the risk of misclassifying retrotransposons as EPRVs, representative plant Ty3/Gypsy LTR retrotransposons from the GyDB database and a *human immunodeficiency virus* (HIV) reverse transcriptase were used as outgroups.

## Results

### Diversity of yam EPRV repeats

Using the CAULIFINDER Branch A protocol, we reconstructed 3,789 EPRV sequences from 86 WGS library datasets representing five *Dioscorea* species. We designated these as yam EPRV repeats (EPRVr) (See supplementary file). The sizes of these reconstructed EPRV sequences ranged from 1,535 bp in *D. praehensilis* to 9,535 bp in *D. dumetorum*. Most were partial sequences mainly comprising of ORF 3-like fragments interspersed with host intergenic DNA (Supplementary Table S2).

Amino acid sequences of the EPRVr RT–RNase H region were clustered using CD-HIT with a 80% identity threshold. Clusters were defined as groups of sequences sharing ≥ 80% amino acid identity and interpreted as putative distinct EPRVr integration lineages. The number of clusters per accession varied among yam samples and *Dioscorea* species. In *D. rotundata* landraces, 13–24 clusters were detected per accession, while interspecific *D. rotundata* hybrids showed a broader range of 8–38 clusters per sample.

Cluster sharing—defined as clusters containing sequences from multiple species at the ≥ 80% identity threshold—was assessed across species. *D. praehensilis* and *D. abyssinica* shared 31 of 32 clusters and jointly shared 25 of the 28 clusters identified in *D. rotundata*. In contrast, *D. dumetorum* shared 6 of its 52 clusters with *D. rotundata*, while *D. alata* shared 14 of its 35 clusters with *D. rotundata* (see Supplementary Fig. S1).

**Fig. 1:**
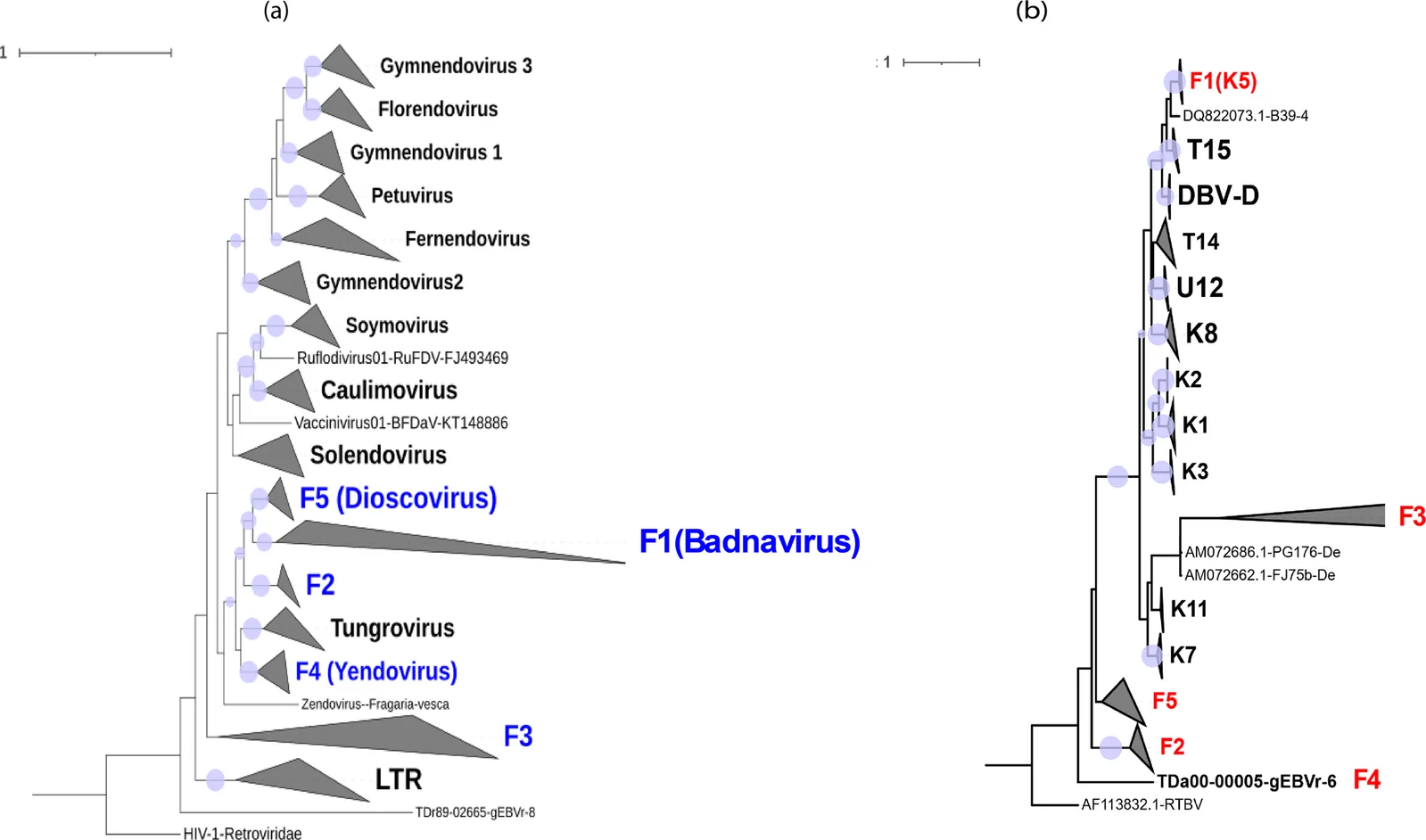
Diversity and evolutionary relationships of yam endogenous pararetrovirus repeats (EPRVrs) determined using maximum-likelihood (ML) phylogenetic analysis based on the RT/RNase H region. Multiple sequence alignments of EPRVrs generated in this study (indicated in bold) and those downloaded from the NCBI database were generated with MAFFT, substitution models were selected with ModelFinder, and branch support was evaluated with 1,000 ultrafast bootstrap replicates. (a) EPRVrs from yams and representative *Caulimoviridae* and other plant endogenous viral groups were analyzed together; yam EPRVrs grouped into F1–F5 (shown in blue). (**b**) Yam EPRVrs were compared to known yam badnavirus phylogenetic groups. In (**a**), the ML tree was rooted with an HIV-1 reverse transcriptase sequence; in (**b**), it was rooted with *Rice tungro bacilliform virus* (genus Badnavirus). Light blue circles indicate branches with > 80% support. EPRVrs identified in this study fell within Group F1-5

Maximum-likelihood phylogenetic analysis of representative EPRVr RT/RNase H amino acid sequences, clustered at 80% sequence identity to select non-redundant representatives, resolved into five well-supported clades (F1–F5; bootstrap support ≥ 80%) (Fig. 1A). Clade F1 clustered with previously characterized yam badnaviruses [29], including badnavirus group K5, which contains *Dioscorea bacilliform RT virus 3* (DBRTV3), originally defined by RT–RNase H sequence similarity [5] (Fig. 1B). This clade was designated Yam Endovirus Repeats. Clade F5 formed a monophyletic group with *Dioscorea nummularia-associated virus* (DNuAV), providing the first evidence of endogenous sequences of the genus *Dioscovirus in D*. *rotundata*. Clade F4 grouped with known endogenous Yendovirus sequences from other plants [17]. In contrast, Clades 2 and 3 did not show close phylogenetic affinity to any established genus within the *Caulimoviridae*, suggesting they represent previously undescribed viral lineages.

### Diversity of yam EPRVs

The CAULIFINDER Branch B protocol detected 673 putative RT/RNase H sequences in *Dioscorea* genomes (Supplementary Table 3). Species-level EPRV diversity (by RT-RNase H at.

**Fig. 2:**
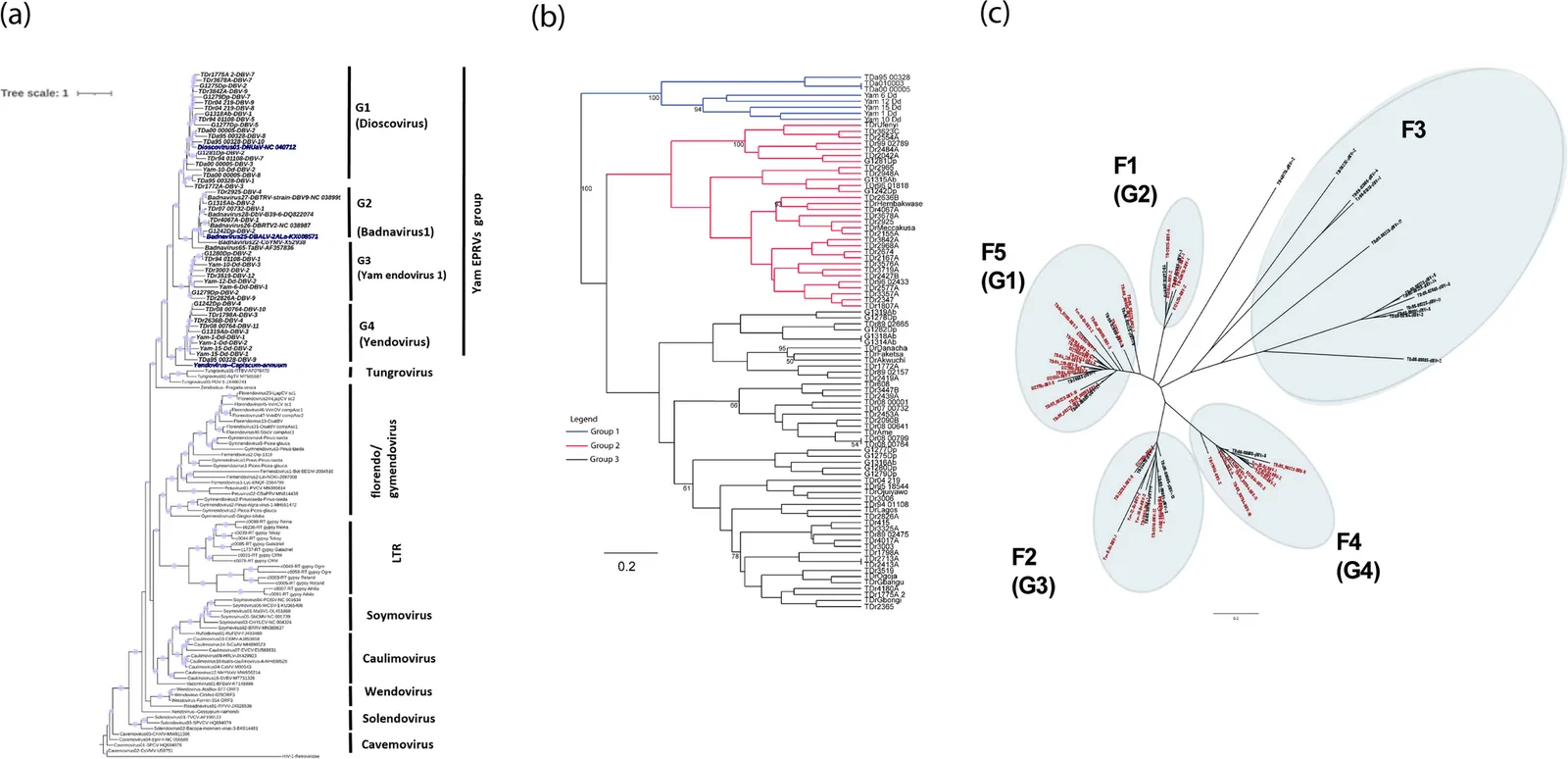
Diversity and phylogenetic relationships of yam EPRVs based on RT/RNase H sequence. Sequence alignments were generated using MAFFT, substitution models were selected using ModelFinder, and phylogenetic trees were inferred using maximum-likelihood (ML) methods with 1,000 ultrafast bootstrap replicates for panels (a) and (c). Bootstrap values for branches with ≥ 80% are indicated at nodes. **(a)** ML phylogenetic analysis of EPRV sequences based on amino acid sequences of the RT/RNase H region, clustering sequences into four major phylogenetic groups (G1–G4). **(b)** Hierarchical clustering of EPRVs from various *Dioscorea* species based on 80% amino acid sequence similarity of the RT/RNase H region. Group 1 (indicated in blue) mainly consists of sequences from *D. alata* and *D. dumetorum*, whereas Groups 2 (indicated in red) and 3 (indicated in black) have more sequences from *D. rotundata*, *D. praehensilis*, and *D. abyssinica*. Branch lengths indicate the degrees of dissimilarity between clusters, not evolutionary distance.**(c)** ML phylogenetic analysis combining EPRV repeat (EPRVr; Branch A) and EPRV (Branch B) sequences based on nucleotide sequences of the RT/RNase H region, presented as an unrooted tree. Sequences identified by the CAULIFINDER Branch A pipeline (repeat-based reconstruction) are indicated in black, and those from Branch B (RT/RNase H–based detection) are indicated in red. Shaded regions indicate major EPRV clusters (F1–F5). The consistent clustering of sequences from both branches supports the reliability of lineage assignments across the two detection approaches

80% sequence similarity), estimated as mean EPRV clusters per accession, was lowest in *D. dumetorum* (2.4), followed by *D. rotundata* (8.1) and *D. alata* (9.0). Using an 80% nucleotide identity threshold, 44 distinct EPRV clusters were defined across all species (Supplementary Table S3). Overall, RT/RNase H sequences of *D. praehensilis* and *D. abyssinica* clustered closely with those in *D. rotundata* (see Supplementary Fig. 1B), a pattern confirmed by hierarchical clustering of the EPRV profiles, which indicate greater similarity between *D. rotundata* with its progenitors, *D. praehensilis* and *D. abyssinica*, than with *D. alata* or *D. dumetorum* (Fig. 2B). Phylogenetic analysis of RT/RNAse H aa sequence revealed four well-defined clades (G1–G4) (Fig. 2A), with bootstrap values ≥ 80%, and sequences in each clade were within 68% to 90% within group sequence identity (see Supplementary Fig. 2).

Clade G3 showed no phylogenetic affinity to any known *Caulimoviridae* reference sequences and was therefore designated Yam Endovirus 1 (YEDV1). Clade G2 clustered with known yam *Badnaviruses*. Clade G1 formed a monophyletic group with *Dioscorea nummularia-associated virus* (DNuAV), the sole recognized member of the genus Dioscovirus (Fig. 2B). As G1 sequences shared < 80% nucleotide identity with DNuAV in the RT/RNase H region, they were designated Dioscorea rotundata dioscovirus (DRDV). A separate lineage in *D. alata* (sharing < 80% identity with both DNuAV and DRDV) was designated Dioscorea alata dioscovirus (DADV) (Fig. 2B). Phylogenetic reconstruction showed that viral lineages reconstructed from EPRVr sequences were more diverse than those inferred from EPRVs (Fig. 2C).

### Genome organization of yam EPRVs

To determine the presence of complete viral genomes, assembled contigs were screened for continuous open reading frames (ORFs) and conserved domain architecture. Using RT/RNase H sequences as anchors, flanking regions were extracted to evaluate viral genome organization. This approach identified two sequence classes: (i) integrated viral fragments flanked by host genomic DNA, and (ii) contiguous EPRV-like sequences without detectable host flanking regions. The latter group included full-length viral-like sequences that structurally resemble episomal members of the *Caulimoviridae* family, as indicated by continuous ORFs and conserved domains. In the absence of host–virus junctions, these cannot be unequivocally classified as endogenous, and their status as integrated or episomal forms remains unresolved.

**Fig. 3:**
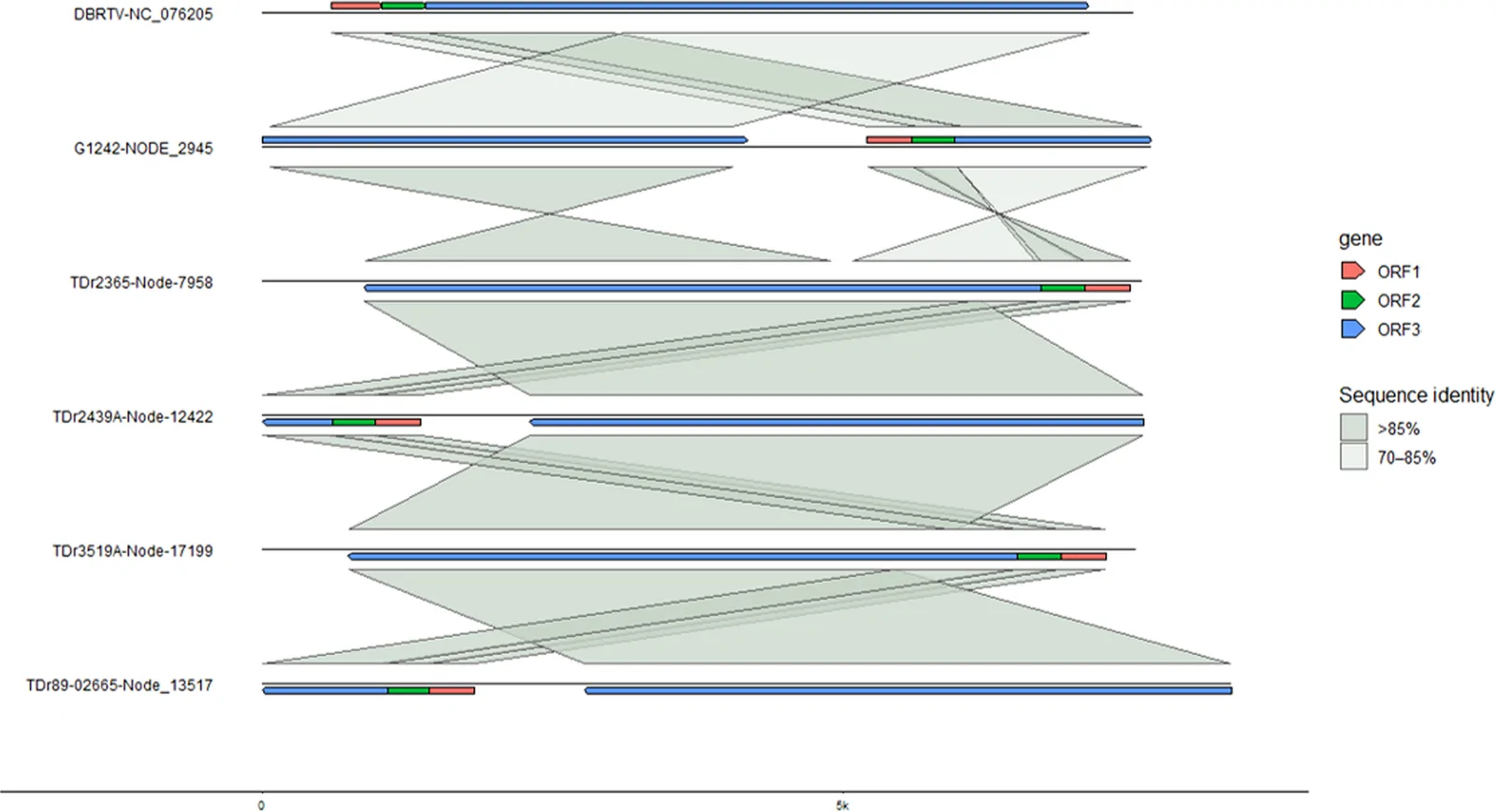
EPRV contigs identified from *D. rotundata* genome assemblies, illustrating partial retention and extensive fragmentation of putative viral ORFs. Schematics of representative assembled contigs containing EPRV-derived sequences are aligned to the corresponding reference badnavirus genome (DBRTV, NC_076205) to depict the heterogeneous and often incomplete nature of endogenous viral integrations. Coloured arrows indicate putative ORFs corresponding to those of the reference badnavirus genome. Grey ribbons connect homologous regions between the reference genome (DBRTV, NC_076205) and EPRV fragments, with ribbon transparency reflecting BLASTn sequence identity

The contiguous EPRV-like sequences (no host flanking regions) were primarily detected within clades G2 (Yam Badnavirus 1) and G1 (Dioscovirus). In clade G2, such sequences were identified in *D. praehensilis* (e.g., contig G1242Dp; Supplementary file). They were also found in *D. rotundata* hybrids and landraces (Fig. 3).Dioscovirus-like sequences were detected exclusively in *D. rotundata* (see Supplementary Fig. 3).

Although assembled as linear contigs, several near ‘full-length’ putative viral genomes lacking detectable host flanking sequences exhibited structural features characteristic of circular *Caulimoviridae* genomes. In the DRDV and DNuAV groups, contigs encoded near-complete polyprotein (ORF 3-like) and transactivator (ORF 4-like) domains that closely resembled those of episomal viruses (Fig. 3). In addition, regulatory elements commonly associated with episomal viral genomes were identified, including plant-like *tRNA*^*Met*^ primer-binding–like sequences and cis-regulatory motifs such as multiple *GATA* boxes and *TATA/TAA* boxes.

For example, in one contig (TDr3447B, NODE_13486), a split ORF 3-like region (ORF 3 A: 2,865 nt; ORF 3B: 2,157 nt) encoded conserved reverse transcriptase–LTR (1,633–2,172 nt), RNase H (2,458–2,841 nt), peptidase (880–1,509 nt), and zinc finger (184–234 nt) domains.

The intergenic region contained an 18-nt *tRNA*^*Met*^-like sequence (TGGTATCAGAGCTTCTGT) at positions 4,489–4,506 nt, together with seven *GATA* boxes and one *TATATAA* box. A similar organization was observed in contig TDr2439A (NODE_12422), which contained a *tRNA*^*Met*^-like motif (TTCTTCTCTTTTGTAAGA) at positions 1,475–1,492 nt and encoded *RT-LTR*, *dUTPase*, *RNase H*,* COG3577*, and *zinc finger* domains on the negative strand. Collectively, these features are consistent with circular viral genome architecture, although biological activity was not assessed.

**Fig. 4:**
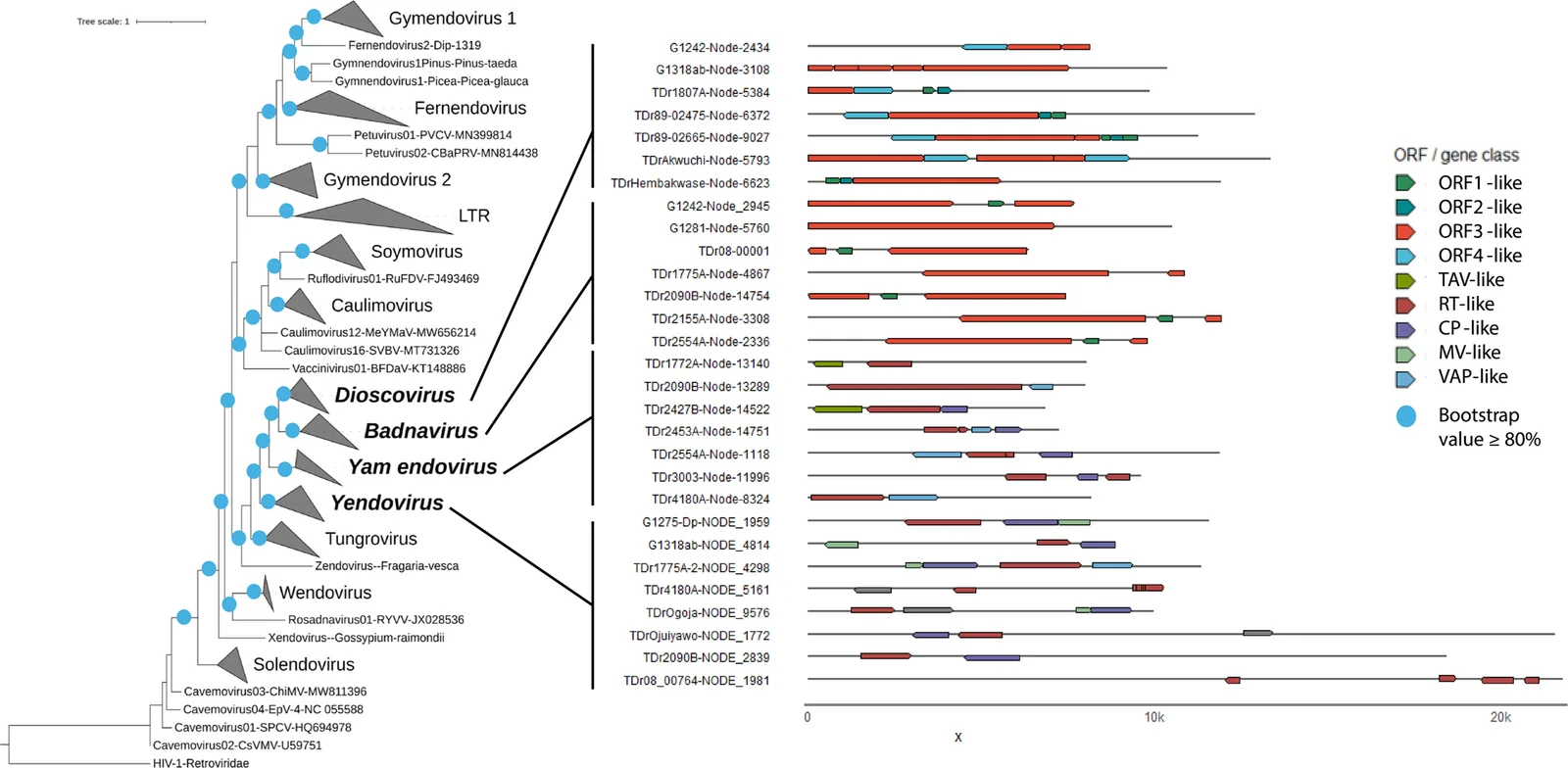
Genomic diversity and organization of yam endogenous pararetroviral elements (EPRVs) identified using CAULIFINDER Branch B. The maximum-likelihood phylogenetic tree is based on the representative EPRVs detected through conserved RT/RNase H domains. Adjacent to the ML tree, representative contigs containing RT/RNase H regions are shown to illustrate the diversity of genomic organization among EPRVs. These contigs were obtained by extending RT/RNase H regions and reconstructing flanking genomic fragments. Coloured blocks denote putative *Caulimoviridae* ORFs or protein coding domains inferred from sequence similarity, highlighting differences in gene content and arrangement across EPRV lineages. Only selected contigs are shown for clarity. Bootstrap support is indicated by blue circles at nodes (≥ 80%)

Based on integration patterns and genome architecture, yam endogenous pararetroviral elements (EPRVs) were classified into four major groups: Yam Badnavirus 1, Yam Endovirus 1, Dioscovirus-like, and Yam Yendovirus. The overall key distinguishing genomic features of each group are summarized below (Fig. 4).

### Yam badnavirus 1

Extension of contigs flanking the RT/RNase H region identified several full-length or nearly complete putative viral sequences within this group. BLASTn analysis revealed > 80% nucleotide identity to known episomal badnaviruses, including *Dioscorea bacilliform AL virus* (DBALV), DBBLV, and DBRTV, with the highest similarity observed in ORF 3, particularly within the RT/RNase H region. These results support their classification as endogenous sequences related to episomal *Dioscorea* bacilliform viruses (DBVs). However, many sequences were fragmented or structurally rearranged, frequently containing insertions of yam-derived DNA that disrupted ORF3-like regions.

### Dioscovirus-like endoviruses

Dioscovirus-like sequences were detected across all screened yam genomes, with over 190 sequences (≥ 80% similarity) recovered from *D. abyssinica*,* D. praehensilis*, and *D. rotundata*. BLASTp analysis showed 74–79% amino acid identity to *Dioscorea nummularia–associated virus* (DNuAV), the reference species for the genus *Dioscovirus*. Although most sequences were fragmented and lacked one or more ORFs, a subset retained clear integration signatures flanked by host DNA and encoded conserved domains characteristic of the family *Caulimoviridae*, including aspartic protease, zinc finger, reverse transcriptase, movement protein, and RNase H domains. Notably, these sequences lacked detectable *tRNA*^*Met*^ primer-binding–like motifs in their genome, in contrast to many Badnavirus-derived EPRVs, suggesting differences in genome organization or integration context. To our knowledge, this represents the first detailed characterization of Dioscovirus-like endogenous sequences in yam, extending previous genome-wide studies that identified related RT-domain sequences across plant species [25].

### Yam yendovirus-like viruses

This well-supported clade (bootstrap support > 80%) comprised more than 130 endogenous EPRV sequences detected across all five *Dioscorea* species. A relatively intact representative sequence (11,565 nt) was identified in *D. praehensilis* (G1275-NODE_1959; associated with the RT/RNase H marker G1275Dp-DBV-1) and was selected as our reference for this group. ORF prediction identified 115 putative ORFs, reflecting extensive coding degeneration rather than functional genome organization. Conserved domain analysis detected caulimoviridae-specific signatures, including reverse transcriptase–LTR, RNase H, pepsin-retropepsin (ORF69), zinc-finger motif (ORF88), and movement protein–like domains (ORF67). Two additional ORFs showed weak similarity to non-viral proteins: ORF106 to a *D. alata* RNase H–like protein and ORF68 to a hypothetical *Piper DNA virus 1* protein. BLASTn analysis of related contigs revealed extensive fragmentation, frequent loss of essential viral domains, numerous premature stop codons, and interruptions by host genomic sequences, consistent with long-term degeneration following ancient integration events.

### Yam endovirus 1

The Yam Endovirus 1 group comprised more than 100 EPRV fragments containing RT/RNase H homology and was detected in all yam species examined. These sequences were highly degraded, characterized by fragmented ORF3-like regions, internal stop codons, and frequent insertions of yam genomic DNA. Conserved domain analysis identified partial matches to *RT-LTR*,* RNase H*,* Zf-CCHC*, and *pepsin-retropepsin* domains, while a subset of sequences showed BLASTp similarity to retroviral capsid-like proteins (up to 67% amino-acid sequence similarity with full query coverage). Collectively, these features are consistent with ancient, fragmented endogenous viral elements with limited or no remaining coding potential.

## Discussion

Our comparative analysis revealed that *D. praehensilis* and *D. abyssinica* share a high proportion of EPRV clusters with *D. rotundata*, whereas *D. dumetorum* and *D. alata* showed lower overlap. This pattern is consistent with the homoploid hybrid origin of white yam [30], derived from hybridization between *D. praehensilis* and *D. abyssinica*. In this context, these findings suggest that EPRVs, particularly Yam Yendovirus (YYeV) and Yam Endovirus 1 (YeDV1), may serve as molecular markers of genomic ancestry and species divergence in yams.

From an evolutionary perspective, the close similarity between yam EPRVs and known episomal viruses reflects repeated historical interactions between *Dioscorea* species and diverse members of the *Caulimoviridae*. Yendovirus- and Endovirus-related sequences were detected in all five *Dioscorea* species, predominantly as fragmented genomes, consistent with the long-term persistence of replication-defective viral elements [18, 31]. However, the presence of related endogenous sequences in multiple species does not, by itself, demonstrate integration prior to species divergence, as similar patterns could also arise through independent integration events from related viral lineages. Resolving the timing of integration warrants future investigation to identify orthologous insertion loci across species.

Comparable patterns have been reported in previous studies of endogenous pararetroviruses, providing a broader context for the diversity observed in yams. In *Dioscorea* spp., De Tomás and Vicient [25] identified Yendovirus-, Badnavirus-, and Dioscovirus-related EPRVs using annotated genome sequences, although their analytical pipeline did not recover the Yam Endovirus 1 group, likely due to extensive sequence fragmentation and degeneration. Beyond *Dioscorea*, Diop et al. [17]. proposed 13 operational taxonomic units (OTUs) for vascular plant EPRVs shaped largely by host phylogeny but did not identify OTUs corresponding to the genus *Dioscovirus*. In this broader comparative context, the absence of Florendovirus-related elements in our analysis—despite their widespread occurrence in flowering plants— is consistent with the view that integration patterns within the *Caulimoviridae* are not uniformly distributed across plant lineages and may instead reflect host-specific histories of virus–host interaction.

Phylogenetic analysis based on an 80% nucleotide identity threshold showed strong concordance between the two detection pipelines (EPRV repeats and EPRVs). Where minor discrepancies occurred, they likely reflected structural variation or copy-number differences influencing detectability rather than conflicting phylogenetic signal. This methodological consistency provides a robust framework for comparing yam EPRV diversity with previous studies.

Seal et al. [10]. reported diverse Badnavirus-related EPRVs within the *Dioscorea cayenensis–rotundata* complex. This study extend this knowledge by demonstrating that yam harbors EPRVs from four distinct genera within the family *Caulimoviridae*. These findings raise two key questions: (i) what is the full extent of EPRV diversity in yam, and (ii) how does these elements affect diagnostics? Although EPRVs are generally considered non-functional due to accumulated mutations following integration [31–33], our results do not exclude the possibility of historical interactions between endogenous and episomal forms. For example, Umber et al. [29] reported four endogenous *Badnavirus* species in yam, which our analysis showed to clustered with previously identified yam badnaviruses. Notably, only Badnavirus- and Dioscovirus-like EPRVs retained near-complete ORF 3 regions, whereas EPRVs from other genera were highly degraded, suggesting selective retention. Similar patterns occur in *Vitis vinifera*, VvinBV (Vitis vinifera B virus) retained coding integrity, unlike VvinAV (Vitis vinifera A virus) and VvinCV (Vitis vinifera C virus) [18], and in banana, where Badnavirus integrations show variable degeneration consistent with co-evolution [31, 34, 35]. Intact EPRVs have been hypothesized to confer resistance or, in some cases, enable episomal reactivation [10], although underlying mechanisms remain unresolved.

Notably, we identified Dioscovirus-like EPRVs in multiple *Dioscorea* species, whereas the only previously reported *Dioscovirus* was episomal and detected exclusively in *D. nummularia* using rolling-circle amplification [36]. By contrast, our genome-wide analysis revealed cryptic Dioscovirus-like integrations across several *Dioscorea* genomes. Although some elements retained relatively intact ORFs, there is currently no evidence that these endogenous sequences are capable of reactivation, and the extensive fragmentation observed in many copies suggests that most are replication defective. Accordingly, the primary significance of these findings lies in their implications for virus detection and diagnostics rather than in demonstrated biological risk.

Geering et al. [18] proposed that some members of the *Caulimoviridae*, such as Florendoviruses, persist as asymptomatic genomic fossils that have co-evolved with their hosts and lost pathogenicity. The detection of episomal *Dioscovirus* in *D. nummularia* and the endogenous Dioscovirus-like contigs identified in other *Dioscorea* species are consistent with this model.

Taken together, this study provides a consolidated view of endogenous pararetroviral diversity across cultivated and wild *Dioscorea* species, revealing clear species-level patterns. *D. rotundata*, *D. praehensilis*, and *D. abyssinica* share the highest diversity and abundance of EPRVs—dominated by Badnavirus-, Yendovirus-, and Endovirus-related elements—consistent with their close evolutionary relationships, whereas *D. alata* and *D. dumetorum* harbor fewer fragmented integrations. The majority of EPRVs across all yam species were structurally degraded, lacking complete viral genomes and essential regulatory motifs, indicating that these elements are replication-defective and biologically inactive. Importantly, no evidence was found to support episomal activity or reactivation potential of any endogenous sequence detected in yams. The extensive sequence similarity between endogenous elements and episomal viruses within conserved RT/RNase H domains explains the limited specificity of existing PCR assays.

## Conclusions

These findings expand the known endogenous viral element (EVE) landscape in *Dioscorea*, demonstrating that yam EPRVs extend well beyond the previously characterized endogenous yam *Badnaviruses*. Notably, the identification of dioscovirus-like elements highlights the power of WGS to uncover cryptic, integrated viral sequences that are not readily detectable using conventional diagnostic methods. Collectively, these results contribute to a deeper understanding of virus–host associations in vegetatively propagated crops and provide a framework for resolving the phylogenetic structure and lineage-specific distribution of EPRVs in yam.

Future investigations on transcriptional activity, epigenetic regulation, and biological significance of EPRVs in yam would help understand the functional relevance of EPRVs, including whether EPRV sequences confer selective advantages, such as interference with related viruses, or have any negative consequences on host or simply neutral genomic features. In addition, analyses of chromosomal distribution and integration patterns across cultivated and wild *Dioscorea* species may offer insights on evolutionary significance to understand persistence and genomic context of EPRVs and ultimately aid in yam germplasm management strategies.

Lastly, the findings of this study have significant implications for yam phytosanitation, diagnostics, and international germplasm exchange. (i) Since most EPRVs in yams are structurally degraded and replication-defective, they are unlikely to pose a direct plant health risk. This reduces concerns that tissue culture or stress could activate badnavirus infections from integrated sequences. For genebanks and breeding programs, this finding clarifies that yam accessions carrying endogenous badnavirus fragments can be maintained and distributed with greater confidence, supporting safer exchange of genetic resources. (ii) The high sequence similarity between endogenous elements and episomal badnaviruses in conserved RT/RNase H regions, which are often targeted by PCR-based diagnostics, may lead to false positives. Current diagnostic protocols should therefore be refined to more accurately distinguish integrated sequences from episomal infections. (iii) Risk assessment frameworks should differentiate between biologically active episomal infections and inactive integrated viral relics and avoid regulatory decisions based solely on detecting inactive endogenous sequences. Overall, these results support a science-based, risk-proportionate approach to yam phytosanitation that facilitate global germplasm exchange while maintaining biosecurity.

## Supplementary Information

Below is the link to the electronic supplementary material.

Supplementary Material 1

Supplementary Material 2

## Data Availability

The sequencing data for the findings presented in this study are available at: https://figshare.com/s/2df935d6c91c65a16fdb.
