# Supplementary material for: Characterization of endogenous pararetroviruses in yam (Dioscorea spp.) genomes revealed four pararetrovirus groups, including a dioscovirus-like lineage: implications for diagnostics and yam germplasm exchange
Source: Arch Virol. 2026 Aug 7;171(9):247. doi: 10.1007/s00705-026-06703-4 (PMC13451355; doi:10.1007/s00705-026-06703-4)
Supplement: Supplementary file 1 [file 705_2026_6703_MOESM2_ESM.docx]

**Supplementary Figure S1.** Diversity and evolutionary relationships of yam endogenous pararetrovirus (EPRV) repeats. (a) Average number of EPRV clusters per sample (±SE) across five *Dioscorea* species. (b) Venn diagram showing shared and unique EPRV clusters, based on 80% amino acid sequence similarity.

**Supplementary Figure S2**. Diversity and phylogenetic relationships of yam endogenous pararetrovirus (EPRV) sequences.Sequence identity comparison as inferred for each phylogenetically distinct group.

**Supplementary Figure S3.** Dioscovirus-like endogenous pararetroviral elements (EPRVs) identified from yam genome assemblies, illustrating partial retention and extensive fragmentation of viral-like ORFs. Diagrams show representative assembled contigs containing Dioscovirus-like EPRV sequences aligned to the corresponding reference virus genome to illustrate the heterogeneous and often incomplete organization of endogenous Dioscovirus-related integrations. For clarity, conserved viral protein domains and regulatory motifs are not shown. Coloured arrows indicate viral-like ORFs corresponding to those of the reference genome. Grey ribbons connect homologous regions between the reference genome and endogenous viral fragments, with ribbon transparency reflecting BLASTn sequence identity. Genome structures are shown for descriptive purposes only and do not imply functional integrity, replication competence, mechanistic integration pathways, or predictive relationships
